# High BRAF Mutation Frequency and Marked Survival Differences in Subgroups According to KRAS/BRAF Mutation Status and Tumor Tissue Availability in a Prospective Population-Based Metastatic Colorectal Cancer Cohort

**DOI:** 10.1371/journal.pone.0131046

**Published:** 2015-06-29

**Authors:** Halfdan Sorbye, Anca Dragomir, Magnus Sundström, Per Pfeiffer, Ulf Thunberg, Monica Bergfors, Kristine Aasebø, Geir Egil Eide, Fredrik Ponten, Camilla Qvortrup, Bengt Glimelius

**Affiliations:** 1 Department of Oncology, Haukeland University Hospital, Bergen, Norway; 2 Department of Pathology, Uppsala University Hospital, Uppsala, Sweden; 3 Department of Immunology, Genetics and Pathology, Uppsala University, Uppsala, Sweden; 4 Department of Oncology, Odense University Hospital, Odense, Denmark; 5 Department of Global Public Health and Primary Care, Lifestyle Epidemiology Group, University of Bergen, Bergen, Norway; 6 Science for Life Laboratory, Uppsala University, Uppsala, Sweden; Ohio State University Medical Center, UNITED STATES

## Abstract

RAS and BRAF mutations impact treatment and prognosis of metastatic colorectal cancer patients (mCRC), but the knowledge is based on trial patients usually not representative for the general cancer population. Patient characteristics, treatment and efficacy according to KRAS, BRAF and MSI status were analyzed in a prospectively collected unselected population-based cohort of 798 non-resectable mCRC patients. The cohort contained many patients with poor performance status (39% PS 2-4) and elderly (37% age>75), groups usually not included in clinical trials. Patients without available tissue micro array (TMA) (42%) had worse prognostic factors and inferior survival (all patients; 7m vs 11m, chemotherapy-treated;12m vs 17m). The 92 patients (21%) with BRAF mutation had a poor prognosis regardless of microsatellite instability, but receipt of 1-2^nd^ chemotherapy was similar to wildtype BRAF patients. Median survival in this cohort varied from 1 month in BRAF mutated patients not given chemotherapy to 26 months in wildtype KRAS/BRAF patients <75 years in good PS. TMA availability, BRAF mutation and KRAS mutation were all independent prognostic factors for survival. The observed 21% BRAF mutation incidence is higher than the previously and repeatedly reported incidence of 5-12% in mCRC. Screening for BRAF mutations before selection of treatment is relevant for many patients, especially outside clinical trials. A BRAF mutation only partly explained the very poor prognosis of many mCRC patients. Survival in unselected metastatic colorectal cancer patients is extremely variable and subgroups have an extremely short survival compared to trial patients. Patients without available TMA had worse prognostic factors and shorter survival, which questions the total generalizability of present TMA studies and implies that we lack information on the biologically worst mCRC cases. Lack of available tissue is an important underexposed issue which introduces sample bias, and this should be recognized more clearly when conclusions are made from translational mCRC studies.

## Introduction

In metastatic colorectal cancer (mCRC) trials, median survival now regularly exceeds 20 months and approaches 30 months [1–4]. Molecular and gene analyses on tumor tissue give prognostic and predictive information. Screening for mutations in RAS and BRAF oncogenes is used in clinical practice and impacts treatment selection. Mutations in the KRAS codon 12 of exon 2 are strongly predictive for lack of benefit from EGFR-inhibition [1–3]. Rare RAS mutations also indicate no benefit from EGFR-inhibition [4]. Mutations in the BRAF oncogene have been found in 5–12% of mCRC patients (S1 Table). The single substitution missense mutation V600E accounts for more than 95% of BRAF mutations. The BRAF mutation (mutBRAF) indicates a poor prognosis with median survival of 10 months in trial patients [2,3,5–10]. The negative prognostic impact of mutBRAF after primary tumor resection in colorectal cancer seems to be restricted to patients with microsatellite stable tumors [11–13].

The molecular studies behind these results are mainly based on tumor specimens from patients included into trials. However, trial patients are selected and usually not representative for the general cancer population. In recent cancer registry data, median survival for mCRC patients was only 10 months, although it had increased compared to earlier years [14–17]. Patients included into trials are from sub-populations with better prognostic factors as they are younger, have better performance status (PS), less co-morbidity and better baseline prognostic factors [18]. Furthermore, population-based studies have shown that approximately one third of mCRC patients do not receive palliative chemotherapy at all [14,15,18,19]. Population-based data on incidence, prognostic and predictive effects of BRAF and KRAS mutations in mCRC patients are therefore needed.

In the present study we analyzed the prognostic and predictive value of BRAF/KRAS mutations and tumor tissue availability in patients with non-resectable mCRC included in a prospective population-based study [18]. This cohort represents an unselected population of all non-resectable mCRC patients in a defined area, including patient groups usually not present in clinical trials and where molecular and clinical data are very scarce; patients with poor PS, elderly patients and patients not given chemotherapy. The aim of the study was: i: To describe the true incidence of BRAF mutations in unselected mCRC patients, ii: To investigate if BRAF mutations alone could explain the very poor prognosis for mCRC patients with poor PS, iii: To characterize patients without available tumor tissue for molecular analyses, to decide generalizability of prior TMA studies, and iv: To study heterogeneity of survival for unselected mCRC patients in comparison to selected trial patients.

## Material and Methods

### Patient cohort

Prospective registration of patients with non-resectable, histologically confirmed mCRC referred to the oncology units of three university hospitals in Scandinavia was performed between October 2003 and August 2006. These hospitals received all oncology department referrals of their administrative area. Uppsala University Hospital in Sweden served a region of 280 000 inhabitants, Odense University Hospital in Denmark 475 000 inhabitants and Haukeland University Hospital in Norway 450 000 inhabitants. Clinical characteristics, blood tests, treatments, response to treatment, progression-free survival and overall survival were recorded. The cohort was originally set up with the intent to study trial inclusion. The cohort was later expanded to also include mCRC patients not seen at the oncology departments using regional cancer registries checking CRC deaths during October 2003-December 2008. Patients were included if initial mCRC diagnosis was between October 2003-August 2006. The present cohort therefore represents an unselected population of all non-resectable mCRC patients in a defined area. Baseline variables were from the patients' first consultation at the oncology department or at the diagnosing health care unit if not referred. Combination chemotherapy was irinotecan or oxaliplatin with a fluoropyrimidine. Written informed consent was given by all participants seen at the clinics. Patient information was anonymized and de-identified prior to analysis. The study was approved by the ethical committees in Norway (Regional Committee for medical and health research ethics-REC West), Sweden (Regional Ethical Committee Uppsala) and Denmark (The Regional Scientific Ethical Committees for Southern Denmark).

### Tissue retrieval and tissue microarray generation

Paraffin embedded tissue blocks of the primary tumor or metastatic lesion were retrieved and corresponding hematoxylin-eosin stained glass slides were examined. Representative parts of the tumor were selected for tissue microarray (TMA) generation and DNA extraction. Tissue cores of 1 mm in diameter were extracted from the original block and transferred to a recipient paraffin block (for TMA construction) or to a clean vial for DNA extraction, after comparison with the marked HE-stained slide. On the glass slide, areas with viable tumor with as little as possible other cells admixed were marked. Care was taken to insure the correct alignment of the glass slide with the paraffin block [20]. TMAs were generated using a TMArrayer and the Beecher Instruments Manual Tissue Arrayer MTA-1.

### Mutation and MSI analysis

DNA was extracted from the tissue cores using Recoverall Total Nucleic Acid Isolation (Ambion, Austin, Texas, USA). KRAS and BRAF pyrosequencing mutational analysis was performed according to the manufacturer’s protocol for the PyroMark Q24 (QIAGEN GmbH, Hilden, Germany) and the use of PCR primers previously described for KRAS codon 12/13 and BRAF codon 600 [21]. Ten ng genomic DNA was used for each PCR reaction. The pyrosequencing analysis was performed as described [22]. Sequencing primer for KRAS codon 12/13 was 5´-AACTTGTGGTAGTTGGAGCT-3´ and for BRAF codon 600 5´-TGATTTTGGTCTAGCTACA-3´. MSI status was determined using MSI Analysis System, version 1.2 (Promega, Madison, WI) with 6 ng genomic DNA and analysis of five mononucleotide repeat markers (BAT25, BAT26, NR-21, NR-24 and MONO-27) on a 3500 genetic analyzer (Applied Biosystems, Fostercity, CA). The samples were denoted MSI-High (MSI-H) if two or more of the five markers showed instability, MSI-Low (MSI-L) if one marker showed instability and microsatellite stable (MSS) if no markers displayed instability.

### Statistics

Group comparisons were performed using the exact chi-square tests for dichotomous or nominal variables, the exact Mann–Whitney tests for ordinal variables, and Student’s t-tests for continuous variables. Binary and ordinal logistic regression was used for dichotomous and ordinal outcome variables, respectively. Results are reported as odds ratios (OR) and 95% confidence intervals (CI). Overall survival time was the interval from the date of non-resectable metastatic disease to the date of death or censored if the patient was alive on February 4th 2014, using the Kaplan–Meier method with log-rank tests for univariate and Cox regression for multivariate analyses. For the multivariate survival analyses, backward stepwise selection of covariates excluding the most non-significant variable at each step according to the likelihood ratio test was used. Variables included in the initial multivariate survival analyses but not shown to be independent factors were: age, gender, smoking habits, study inclusion and location of metastases. Results are reported as hazard ratios (HR) and 95% confidence interval (CI). All analyses were performed with the statistical program SPSS v22. All statistical tests were two tailed, using the 5% significance level.

## Results

**Fig 1:**
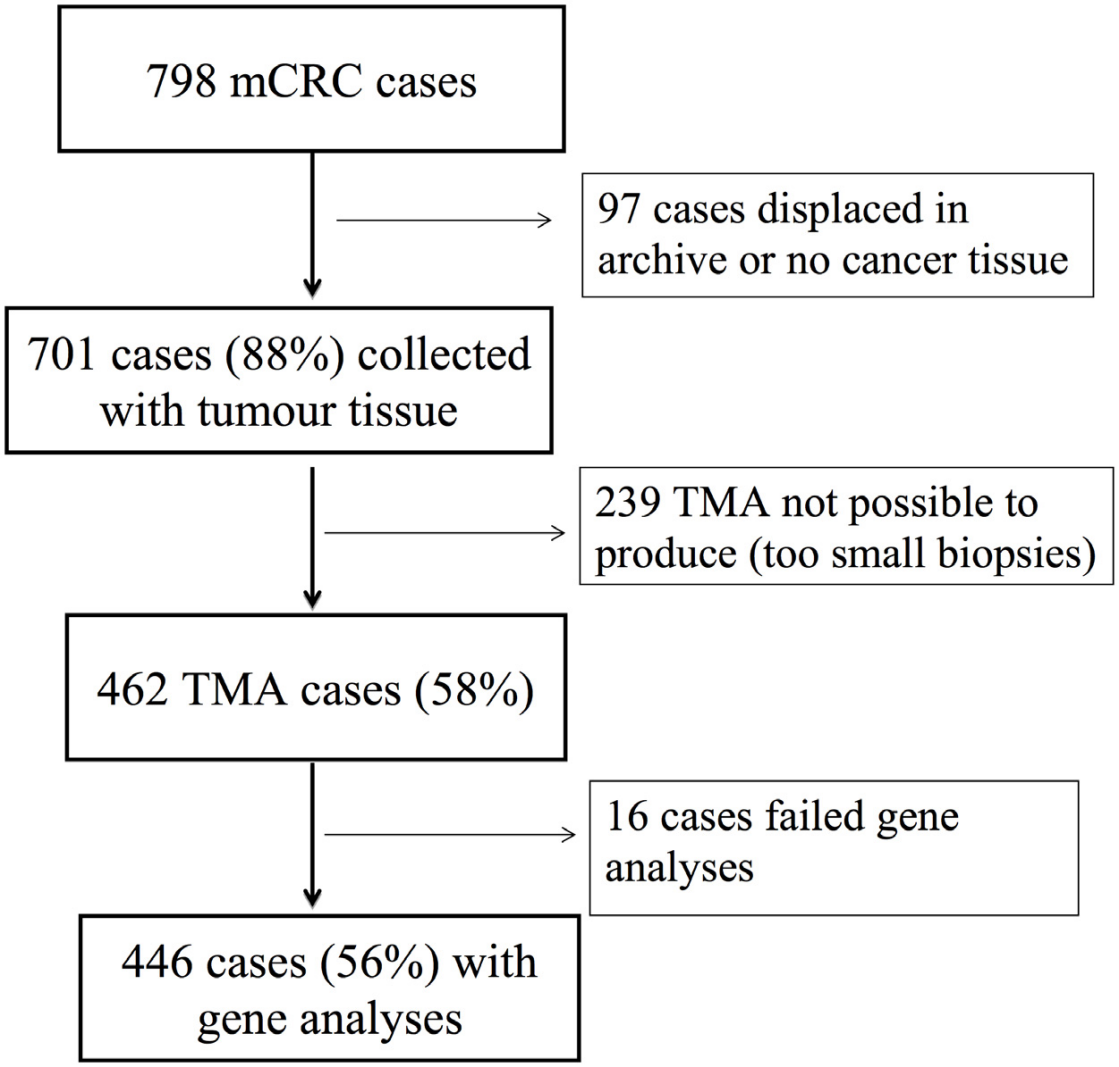
Collection of tumor blocks and tissue micro array (TMA) availability in a population-base cohort of 798 cases with metastatic colorectal cancer (mCRC).

The study finally included 798 patients after exclusion of eleven patients due to re-evaluation or autopsy confirming other cancer than CRC (6 patients) or non-metastatic disease (5 patients). Forty-nine patients (6%) were never seen at the oncology departments. At last follow-up on February 2014, 24 patients (3%) were alive. First-line chemotherapy was given to 456 patients (57%), 77% of these received combination chemotherapy. Bevacizumab was given to 50 patients (11%) and EGFR-inhibitor to 111 patients (24%). Median survival (95%CI) for all 798 patients was 9 months (7.8–10.2), 15 months (13.4–16.6) for chemotherapy-treated patients and 2 months (1.5–2.5) for patients given best supportive care only. Poor PS or high age were the main reasons for not receiving chemotherapy. Tumor blocks containing tumor tissue were collected in 701 (88%) of 798 cases (Fig 1). For the remaining 97 cases, the tumor tissue blocks did either not contain cancer tissue (typically severe dysplasia in a colon/rectum tumor biopsy with metastatic disease and elevated CEA) or the blocks were displaced in the archive. TMA was possible to produce from 462 of 701 collected cases (58% of initial 798 cases), whereas the remaining 239 patients had biopsies being too small or containing necrotic tissue. TMA was almost exclusively made from the primary tumor; 5 were made from metastatic lesions. Gene analyses for BRAF failed in 16 patients and eventually 446 patients had reliable BRAF analyses and 442 patients had both BRAF and KRAS analyses. Among patients with evaluable BRAF status, 277 patients (62%) received chemotherapy.

### TMA

**Fig 2:**
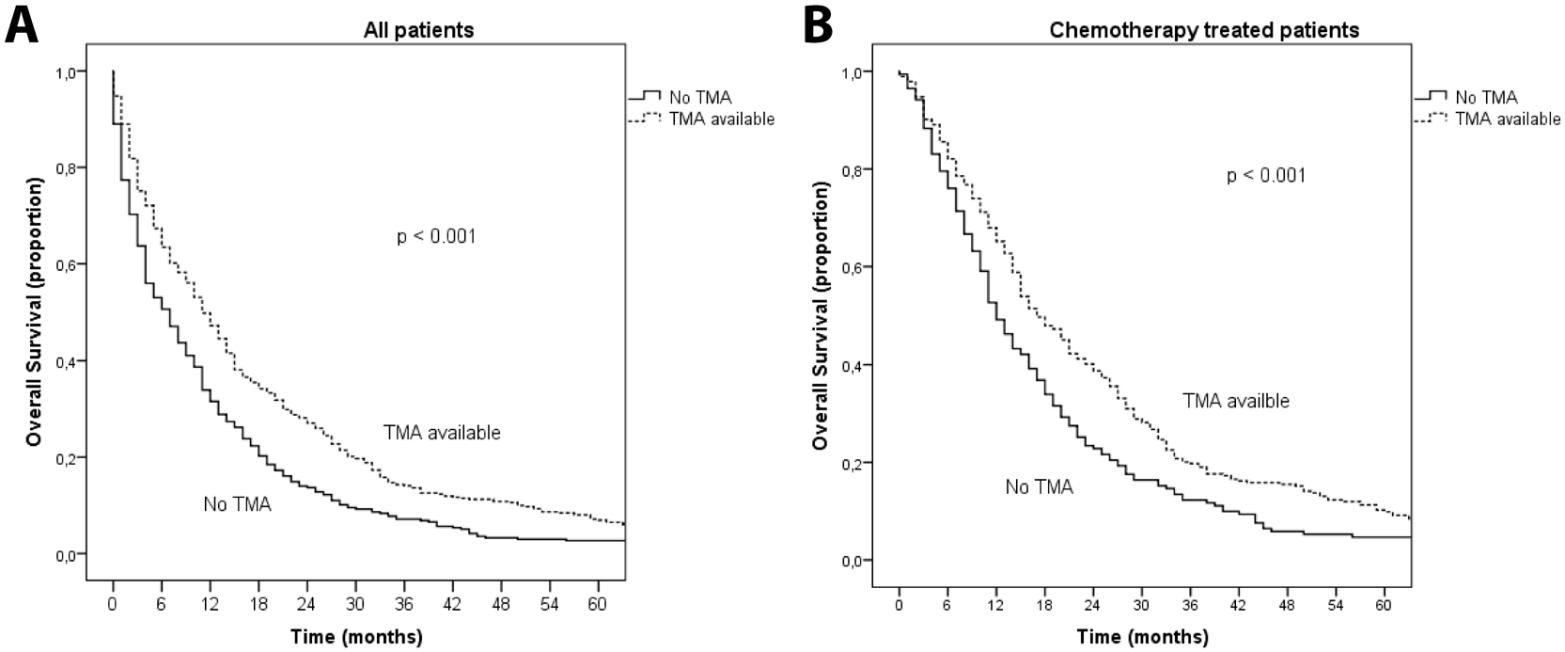
Overall survival in a population-based cohort of 798 metastatic colorectal cancer patients comparing patients with and without available cancer tissue for tissue microarray (TMA) analyses (Kaplan-Meier curves with log-rank test): **A.** Survival for all patients according to TMA availability (n = 798). Median survival for patients with no TMA was 7m vs 11m for patients with TMA available, p<0.001. **B**. Survival for patients treated with 1-line chemotherapy according to TMA availability (n = 457). Median survival for patients with no TMA was 12m vs 17m for patients with TMA available, p<0.001.

Patient characteristics and treatment were significantly different in patients with available TMA, compared to patients where TMA was not possible to produce (Table 1). Patients without available TMA had worse prognostic factors; higher age, primary tumor less resected, more often synchronous metastatic disease, worse PS, more metastatic sites, more often weight loss and anorexia and more often abnormal baseline prognostic blood tests with elevated leucocytes, platelets and alkaline phosphatase [23]. Independent baseline characteristics by logistic regression for patients with available TMA vs. non-available TMA were metastatic sites >1: (OR 0.5, 95% CI 0.4–0.7, p<0.001), synchronous disease (OR 0.5, 95%CI 0.3–0.8, p<0.001), colonic primary (OR 3.6, 95%CI 2.2–6.0, p<0.001) and distant metastatic lymph nodes (OR 2.4, 95%CI 1.3–4.2, p = 0.002). Patients without available TMA less often received chemotherapy (50% vs 63%, p< 0.001) and when given, combination chemotherapy was less used (39% vs 48%, p = 0.0014). Furthermore, fewer were included into clinical trials and fewer received secondary surgery (Table 1). Response rate to 1^st^-line chemotherapy was similar, whereas progression-free survival (PFS) was borderline significantly shorter (6.3 vs 8.0 months, p = 0.06). Median survival was inferior in patients without available TMA compared to patients with TMA in all patients (7 vs 11 months, log-rank p<0.001) and in chemotherapy-treated patients (12 vs 17 months, log-rank p<0.001) (Fig 2). Availability of tissue for TMA was an independent prognostic factor for survival in the multivariate analysis (p = 0.01, Table 2).

**Table 1:** Patient characteristics and treatment comparing mCRC patients with tumor tissue available for tissue micro array (TMA) with patients where tumor tissue was not available for TMA.

Characteristic	TMA(n = 462)n (%)	No TMA (n = 336) n (%)	Exact p-value	Missing
Age in years, median	63	64	ns	
Age				
< 60 years	111 (24)	70 (21)	0.041	
60–75 years	195 (42)	123 (36)		
>75 years	156 (34)	143 (43)		
Sex				
(male)	229 (50)	189 (56)		
(female)	233 (50)	147 (44)	ns (0.06)	
Primary tumor resected	421 (91)	154 (46)	< 0.001	
Adjuvant chemotherapy	68 (15)	20 (6)	< 0.001	1
Synchronous metastases	243 (53)	238 (71)	< 0.001	
Performance status				
0	165 (36)	85 (26)	< 0.001	3
1	145 (31)	88 (26)		
2	85 (18)	79 (24)		
3–4	67 (15)	81 (24)		
Rectal primary	118 (26)	150 (45)	< 0.001	
Colonic primary	344 (74)	186 (55)		
Metastatic organs = 1	193 (42)	80 (24)	< 0.001	
Liver metastases	293 (63)	226 (67)	ns	
Lung metastases	115 (25)	103 (31)	ns (0.07)	
Lymph nodes metastases	135 (29)	77 (23)	0.047	
Peritoneal metastases	88 (19)	59 (18)	ns	
Co-morbidity	255 (56)	199 (60)	ns	9
Weight loss > 10%	52 (12)	65 (21)	< 0.001	71
Anorexia	140 (32)	152 (49)	< 0.001	66
WBC >10 x 10^9^/l	97 (23)	105 (32)	0.003	42
Hemoglobin < 10 g/dL	65 (15)	86 (26)	< 0.001	30
Platelet count> 400 x 10^9^/l	105 (26)	102 (34)	0.037	97
ALP > 3 UNL	55 (14)	65 (22)	0.004	95
1-line chemotherapy	289 (63)	168 (50)	< 0.001	
1-line combination chemotherapy	219 (48)	130 (39)	0.014	1
2-line chemotherapy	168 (36)	86 (26)	0.002	2
3-line chemotherapy	75 (16)	40 (12)	ns (0.08)	2
1-line response rate CR/PR PD	107 (42) 45 (18)	63 (42) 35 (23)	ns	52
PFS 1-line, median 95%CI	8 m (7.3–8.6)	6.3 m (5.0–7.6)	ns (0.06)	
Included in 1-line trial	114 (25)	55 (16)	0.005	1
Secondary surgery	37 (8)	13 (4)	0.016	1
Best supportive care	146 (32)	147 (44)	< 0.001	2
All patients (n = 798), median survival (95% CI)	11 m (9.4–12.6)	7 m (5.3–8.7)	< 0.001	
Chemotherapy treated patients (n = 461), median survival (95% CI)	17 m (14.5–19.5)	12 m (10.1–13.9)	<0.001	

Metastases = at time of diagnosis of metastatic disease. Exact p-value = chi-square test except log-rank for survival. Synchronous disease = within 6 months after initial diagnose. Abbreviations: m = months, WBC; White blood cell count, ALP; alkaline phosphatase, UNL; Upper normal limit, CR; complete response, PR; partial response, PD; progressive disease, PFS; progression-free survival, ns; not significant.

**Table 2:** Multivariate analyses for overall survival in an unselected cohort with metastatic colorectal cancer.

	In all patients Final model, n = 637			In patients with TMA Final mode, n = 385		
Variables	HR	95%CI	p-value	HR	95%CI	p-value
No TMA	1.25	1.05–1.47	0.01		Not included in model	
PS >1	1.93	1.58–2.35	<0.001	1.77	1.37–2.28	<0.001
Liver site	1.41	1.18–1.70	<0.001	1.30	1.04–1.64	0.023
No 1-line chemo	2.62	2.13–3.22	<0.001	2.96	2.27–3.87	<0.001
No secondary surgery	3.87	2.65–5.65	<0.001	3.80	2.35–6.12	<0.001
ALP > 3 UNL	1.39	1.17–1.65	<0.001	1.84	1.46–2.31	<0.001
Comorbidity present	1.28	1.08–1.51	0.005			
Metastatic site >1	1.41	1.18–1.67	<0.001			
Colon primary	1.28	1.07–1.52	0.008			
Synchronous metastases				1.32	1.06–1.64	0.012
No resection of primary				1.68	1.06–2.65	0.037
BRAF mutation	Not included in model.			2.53	1.88–3.40	<0.001
KRAS mutation	Not included in model.			1.34	1.06–1.69	0.015

HR = hazard ratio, TMA = tissue micro array; PS = performance status, ALP = alkaline phosphatase, UNL; Upper normal limit. For the multivariate survival analysis, backward stepwise selection of covariates excluding the most non-significant variable at each step according to the likelihood ratio test was used

### BRAF mutations

**Fig 3:**
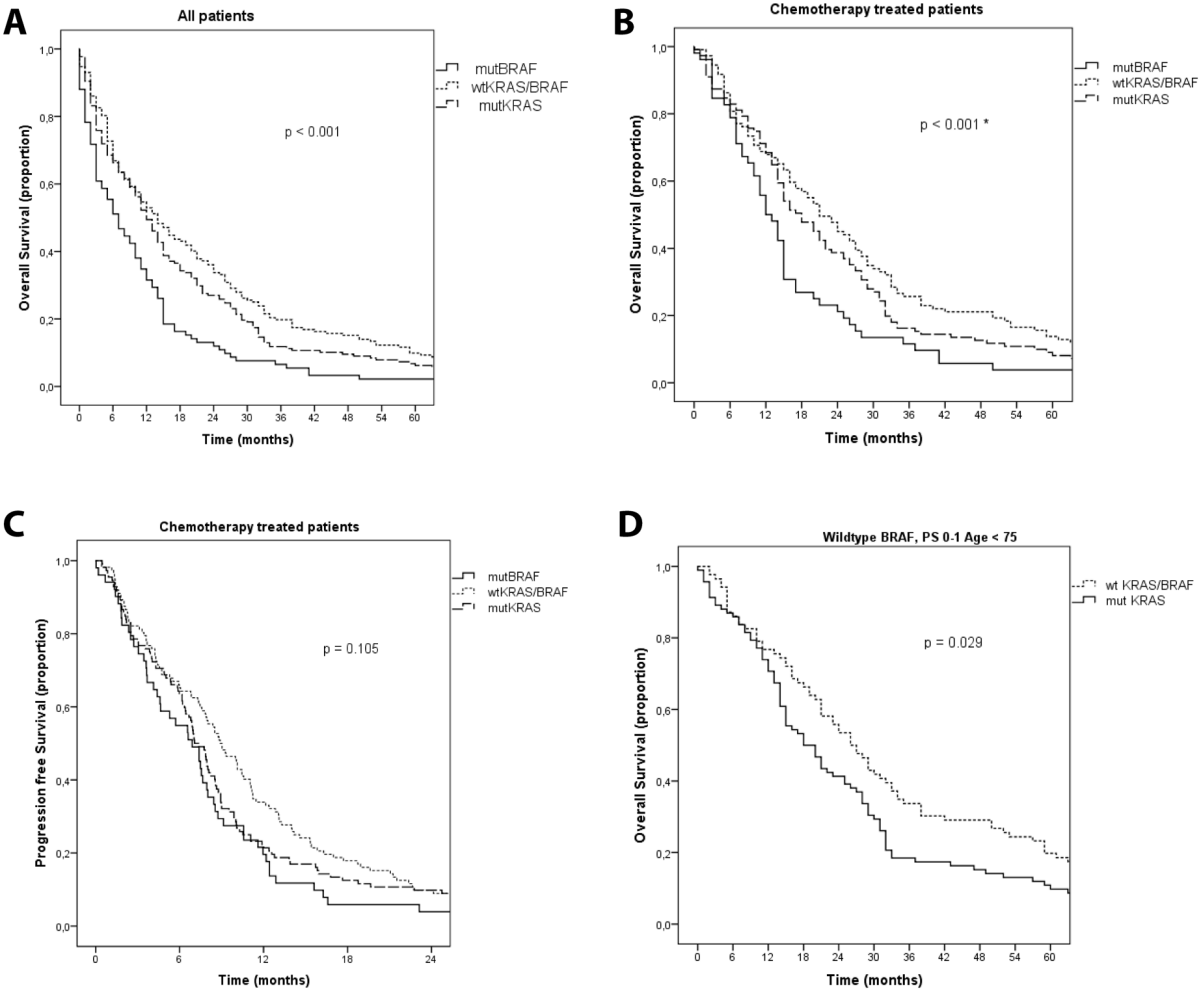
Survival in a population-based cohort of metastatic colorectal cancer patients with available tumor material for analyses of mutation status (mutBRAF, mutKRAS, wtKRAS/BRAF) (Kaplan-Meier curves with log-rank test). **A**. Overall survival for all patients according to mutational status (n = 442). Median survival was 7 m for patients with mutBRAF, 12 m if mutKRAS and 14 m if wtKRAS/BRAF. *mutBRAF significantly different from wtKRAS/BRAF and mutKRAS. **B**. Overall survival in patients receiving chemotherapy according to mutational status (n = 275). Median survival was 12 m for mutBRAF, 18 m for mutKRAS and 21 m for wtKRAS/BRAF. *mutBRAF significantly different from wtKRAS/BRAF and mutKRAS. **C**. Progression free survival in patients receiving chemotherapy according to mutational status (n = 275), Median progression-free survival was 6.9 m for patients with mutBRAF, 7.1 m if mutKRAS and 8.9 m if wtKRAS/BRAF. **D.** Overall survival patients in good performance status (PS 0–1) and age< 75, comparing patients with wtKRAS/BRAF vs mutKRAS tumors (n = 178). Median survival was 18 m for patients with mutKRAS and 26 m for patients with wtKRAS/BRAF. Two patients with BRAF mutation status given chemotherapy were missing KRAS analyses.

A V600E BRAF mutation (mutBRAF) was seen in 92 (20.6%) of 446 patients with gene analyses. In the wtKRAS population (n = 264), 35% were mutBRAF. MutBRAF tumors compared to wtBRAF tumors were more frequent in right colon (58% vs 26%), and less often in sigmoideum (13% vs 35%) and rectum (3% vs 29%). Patients with mutBRAF were more often women and had more often anorexia and weight loss (Table 3). Smoking was not associated with mutBRAF. Independent baseline characteristics for mutBRAF patients compared to wtBRAF patients were; colonic primary (OR 28.5, 95%CI 3.8–216, p<0.001) distant lymph node metastases (OR 2.4, 95%CI 1.2–4.8, p = 0.013), liver metastases (OR 0.4, 95%CI 0.2–0.8, p = 0.007) and weight loss (OR 3.6, 95%CI 1.4–9.2, p = 0.021). MutBRAF patients received 1^st^ and 2^nd^ line chemotherapy as often as wtBRAF patients, but fewer received 3^rd^ and 4^th^-line chemotherapy and secondary surgery (Table 4). Response rate to chemotherapy was similar, whereas PFS was shorter (p = 0.06). Median survival was significantly shorter for mutBRAF patients compared to wtBRAF considering all patients (7 vs 13 months), chemotherapy-treated (12 vs 20 months) and patients receiving best supportive care only (1 vs 4 months) (Table 4, Fig 3). mutBRAF was an independent prognostic factor for survival (p<0.001, Table 2). For patients with good PS and age<75, median survival was 14 months for patients with mutBRAF vs 22 months for those with wtBRAF tumors (p = 0.029).

**Table 3:** BRAF mutations according to baseline characteristics of patients with material available for tissue micro array (TMA) and gene analyses (n = 446).

Characteristic	Patients with mutBRAF n	Frequency of mutBRAF %	mutBRAF vs. wtBRAF p-value	Missing
All patients (n = 446)	92	20.6		
Age in years, median	66			
Age				
< 60 years	19	18	ns	
60–75 years	38	20		
>75 years	35	23		
Sex				
male	34	15		
female	58	26	0.007	
Smoker	23	22	ns	38
Primary tumor resected	88	21	ns	
Adjuvant chemotherapy	13	19	ns	
Synchronous metastases	55	17	ns (0.08)	
Performance status				
0	28	18	ns	
1	25	18		
2	22	26		
3–4	17	27		
PS 2–4 and age < 75	18	24	ns	
Rectal primary	3	3	< 0.001	
Colonic primary	89	27		
Liver metastases	42	15	< 0.001	
Lung metastases	17	15	ns	
Lymph nodes metastases	40	31	0.001	
Peritoneal metastases	22	26	ns	
Metastatic organs = 1	40	21	ns	
Comorbidity	56	23	ns	5
Weight loss > 10%	48	39	0.001	36
Anorexia	38	30	0.008	34
WBC >10 x 10^9^ /l	23	19	ns	29
Hemoglobin < 10 g/dL	13	21	ns	20
Platelet count > 400 x 10^9^ /l	25	19	ns	59
ALP > 3 UNL	9	16	ns	55

Metastases/comorbidity = at time of diagnosis of metastatic disease. P-value = exact p-value by Chi-square test. Synchronous disease = within 6 months after initial diagnose. Abbreviations: WBC; White blood cell count, ALP; alkaline phosphatase, UNL; Upper normal limit, PS; performance status.

**Table 4:** Treatment and treatment results for unselected metastatic colorectal cancer patients according to BRAF/KRAS gene analyses (446 cases with BRAF analyses, 442 cases with both BRAF and KRAS analyses).

Characteristic	All patients n (%)	BRAF mutation n (%)	BRAF wild-type n (%)	p-value	KRAS mutation n (%)	KRAS/ BRAFwild type. n (%)	p-value
All patients		92 (20.6)	354 (79.4)		178	172	
1-line chemotherapy	277 (62)	52 (57)	225 (64)	ns	112 (63)	112 (65)	ns
1-line combination chemotherapy	210 (47)	39 (42)	171 (48)	ns	87 (49)	83 (49)	ns
2-line chemotherapy	160 (36)	28 (30)	132 (37)	ns	65 (37)	66 (39)	ns
3-line chemotherapy	71 (16)	5 (5)	66 (19)	0.002	34 (19)	31 (18)	ns
4-line chemotherapy	27 (6)	1(1)	26 (7)	0.025	10 (6)	16 (9)	ns
Response rate 1-line							ns
CR/PR	102 (42)	17 (37)	85 (43)	ns	35 (37)	50 (49)	(0.07)
PD	45 (18)	10 (22)	35 (18)		15 (16)	20 (20)	
PFS 1-line, median in months (95% CI)	7.9 (7.1–8.6)	6.9 (4.7–9.1)	8.0 (7.2–8.9)	ns (0.06)	7.1 (6.2–7.9)	8.9 (7.1–10.7)	ns
Response rate 2-line							
CR/PR	24 (16)	2 (8)	22 (18)	ns	10 (16)	11 (19)	ns
PD	54 (37)	8 (31)	46 (38)		23 (37)	23 (40)	
PFS 2-line, median in months (95% CI)	4.5 (3.5–5.5)	3.9 (1.6–6.2)	4.6 (3.4–5.8)	ns	4.8 (3.1–6.5)	4.4 (2.0–6.7)	ns
Response rate 3-line							ns
CR/PR	6 (10)	0 (0)	6 (11)	ns	2 (7)	4 (17)	(0.08)
PD	19 (33)	2 (50)	17 (32)		13(45)	4 (17)	
PFS 3-line,median in months (95% CI)	4.3 (2.4–6.2)	2.3 (0.0–5.5)	4.3 (2.5–6.2)	ns	3.0 (2.2–3.7)	5.3 (4.0–6.6)	0.014
Secondary surgery	33 (7)	1 (1)	32 (9)	0.012	14 (8)	18 (11)	ns
BSC all patients	143 (32)	34 (37)	109 (31)	ns	58 (33)	48 (28)	ns
BSC due to poor PS < 75 years of age	29 (10)	6 (11)	23 (10)	ns	13 (11)	9 (8)	ns
All patients, survival median (95% CI)	11 m (9.3–12.7)	7 m (4.2–9.8)	13 m (11.1–14.9)	<0.001	12 m (9.8–14.2)	14 m (10.5–17.5)	ns (0.11)
Chemotherapy- treated patients, median survival.	17 m (14.4–19.6)	12 m (9.0–15.0)	20 m (16.4–23.6)	0.002	18 m (14.2–21.8)	21 m (16.4–25.7)	ns
BSC treated patients, median survival.	4 m (3.0–5.0)	1 m (0.2–1.8)	4 m (2.7–5.3)	<0.001	4 m (2.8–5.1)	5 m (2.0–8.0)	ns
Patients with PS 0–1, age < 75, median survival (n = 178)	21 m (17.4–24.6)	14 m (10.4–17.6)	22 m (18–26)	0.002	18 m (12.9–23.1)	26 m (21.0–31.0)	0.029
Patients with PS 2–4 and age <75, median survival (n = 57)	5 m (3.0–7.0)	2 m (0–4.1)	6 m (4.7–7.3)	0.001	5 m (2.6–7.4)	6 m (2.3–9.7)	0.037

Abbreviations: CR; complete response, PR; partial response, PD; progressive disease, PFS; progression-free survival, PS; performance status, BSC; best supportive care, ns; not significant, m; months. Evaluable patients with KRAS/BRAF analyses were n = 275 for responses/PFS on 1st-line chemotherapy (51 mutBRAF, 112 mutKRAS, 112 wtKRAS/BRAF), n = 157 for responses/PFS on 2nd-line chemotherapy (26 mutBRAF, 65 mutKRAS, 65 wtKRAS/BRAF) and n = 60 for responses/PFS on 3rd-line chemotherapy (4 mutBRAF).

### KRAS mutations

Among wtBRAF tumors, 178 (51%) had a KRAS mutation (mutKRAS) and 172 (49%) patients were wildtype KRAS, i.e. wtKRAS/BRAF. Patients with wtKRAS/BRAF tumors had, compared to patients with mutKRAS tumors, more frequently the primary tumor in sigmoideum (40% vs 30%) and rectum (35% vs 24%), and less in right colon (16% vs 37%). Other baseline and treatment characteristics were similar between mutKRAS and wtKRAS/BRAF patients, except that mutKRAS patients less often received 3^rd^-line chemotherapy (Table 4). In wtKRAS/BRAF patients we found longer PFS in 3^rd^-line treatment. Median survival was similar regardless of treatment. However in patients aged<75 years with good PS, survival was significantly longer for wtKRAS/BRAF patients (26 vs 18 months) (Table 4, Fig 3). mutKRAS was an independent prognostic factor for survival (p = 0.015, Table 2).

### MSI

Microsatellite instability (MSI) was successfully analyzed in 91/92 mutBRAF tumors. Thirty (33%) tumors were MSI-H, 59 MSS and 2 MSI-L. MSI-L was further grouped together with MSS. In mutBRAF tumors, MSI-H compared to MSS tumors were significantly more often located in right colon (80% vs 48%), in elderly patients (57% vs 28%), had less metastases to lung (7% vs 25%) and liver (27% vs 56%). Median OS (95%CI) was in patients with mutBRAF tumors; 5 months (1.8–8.2) for MSI-H and 7 months (2.7–11.3) for MSS (p = 0.21). Among mutBRAF patients given chemotherapy (n = 51), median survival was 8 months (4.1–11.9) for MSI-H patients vs 14 months (12.3–15.7) for MSS patients (p = 0.21). PFS on 1-line chemotherapy was 6.4 months for MSI-H and 7.4 months for MSS patients and not significantly different.

## Discussion

### BRAF mutation

The observed 21% mutBRAF incidence is clearly higher than the previously and repeatedly reported incidence of 5–12% in mCRC (S1 Table). Screening for BRAF mutations before selection of treatment strategy is therefore relevant for many patients, especially in patients cared for outside clinical trials. As our study reveals that patients without available TMA have a much worse prognosis and poor patient characteristics, one could speculate that many of these patients could harbor a BRAF mutation. The true incidence of mutBRAF in the general mCRC population might therefore be even higher than the observed 21%. New treatment options for patients with mutBRAF are evolving as use of FOLFOXIRI in first-line treatment seems to increase survival and combinations of EGFR-inhibitors and BRAF/MEK-inhibitors are promising [24,25]. If mutBRAF is more frequent than anticipated, upfront BRAF testing before 1^st^-line palliative chemotherapy becomes increasingly relevant. Ethnic differences in mutBRAF incidences have been reported [26]. However, mutBRAF does not seem to have a higher incidence in Scandinavians as among the 56 Nordic 7 trial patients in this cohort [2], the incidence of mutBRAF was 12% as in other populations. Although the frequency of mutBRAF in CRC in the Cancer Genome Atlas Network was only 3% [27], population-based studies on mainly stage II-III colorectal or colon cancer patients have found a 12–28% mutBRAF incidence [28–31]. MutBRAF is mainly found in colon tumors, and the relative distribution of colon vs rectal primary in mCRC studies could affect the reported incidence of mutBRAF. The incidence of mutBRAF was 27% in colonic primaries in our study, still higher than the 10–18% reported by other mCRC studies (S1 Table). The BRAF assay used in our study has a 2–5% limit of detection of mutated DNA and is more sensitive than Sanger sequencing. Many different assays are used among prior stage IV CRC studies, but still all report mutBRAF frequencies ranging from 5–12% (S1 Table). We thus believe the higher frequency observed has more to do with the unselected cohort than the method used. Clinical and molecular data based on trial patients alone will not include data on subgroups with poor performance status, elderly and non-treated patients. The present cohort included patients with poor performance status (39% PS 2–4) and elderly (37% age >75), groups underrepresented in clinical trials. Our results, which show a considerable discrepancy in the frequency of BRAF mutations among mCRC patients compared with results from prospective clinical trials, clearly underline the continuous need for observational cohort studies/studies based on registries.

As in prior studies, patients with mutBRAF tumors had more often proximal colon tumors, higher age, female gender, poor PS, multiple metastatic sites, distant lymph node metastasis and synchronous disease but less often lung metastases [6,7,10,32–36]. Two novel observations in our study are that patients with mutBRAF tumors more often had weight loss and anorexia, whereas baseline prognostic serum markers as elevated alkaline phosphatase were not associated with mutBRAF. Different underlying mechanisms may therefore lie behind the prognostic role of these markers. Although a higher incidence of mutBRAF has been reported among smokers and a causal relationship suggested [37,38], we did not observe this similar to a recent study [29]. In our study, patients with mutBRAF tumors received 1^st^ and 2^nd^ line therapy comparable to wtBRAF patients as prior observed [10]. However, they received significantly less 3^rd^ line chemotherapy and only one patient had secondary surgery. mutBRAF tumors are only present in 0–2% in mCRC lung or liver resections [33,39–41]. There seems to be a progressive selection of patients with mutBRAF; 21% or more in the general population as reported here for the first time, 5–12% in trial patients and 0–2% in patients undergoing liver or lung resections. Response to chemotherapy in mutBRAF patients was similar and PFS on 1st-line chemotherapy was only borderline significantly worse. Overall survival was significantly shorter in mutBRAF patients, but varied from 1 month in patients receiving only best supportive care to 14 months in PS 0–1 patients younger than 75 years receiving chemotherapy. Most prior studies have shown a shorter PFS for mutBRAF patients [1,2,5,9,42,43], but some report similar PFS [3,21,44]. Trial patients with mutBRAF have repeatedly had a shorter median survival, usually around 10 months (S1 Table). Initially we hypothesized that mutBRAF could explain the very poor prognosis for many subgroups of mCRC, e.g. in young patients with poor PS and not given chemotherapy. However, as mutBRAF only tended to be more frequent among these patient groups, mutBRAF can only partly explain the very poor prognosis among mCRC patient subgroups. Other candidate genes and molecular factors must be sought. In our study we found that 33% of mutBRAF tumors were MSI-H, which is in accordance with the 29% observed by others [33]. An adverse prognosis for CRC (mainly stages I-III) has been reported with presence of MSS with mutBRAF, but not for MSI-H [11–13]. In our study MSI-status did not affect prognosis in mutBRAF stage IV CRC patients, in accordance with other studies [9,35].

### KRAS mutations, wildtype KRAS/BRAF

It has been recommended that mCRC cohorts should be divided into 3 groups: mutBRAF, mut(K)RAS and wtBRAF/(K)RAS [3,5,8,10]. In most studies KRAS mutations have not been prognostic. However, the comparisons have often been done with a mixed wtKRAS population including mutBRAF. In our study, survival was similar comparing all wtKRAS/BRAF patients to all mutKRAS patients, regardless of treatment. However, in patients aged<75 years with good PS, being the target group for intensive chemotherapy, survival was significantly longer for wtKRAS/BRAF patients (26m vs 18m). Other groups have also found an inferior survival for mutKRAS compared to wtKRAS/BRAF [5,8,10]. Maugham *et al*, found a median survival of 14.4 months in mutKRAS mCRC patients, compared to 20.1 months in wtKRAS/BRAF patients [5]. Our observed survival of 26 months in a fit mCRC population (similar to trial patients) is similar to a recent study, where median survival was 28 months in wtRAS/BRAF patients [3].

### TMA

In the present study, we could produce TMA from 462 patients (58%) of initially 798 cases, and BRAF analyses for 446 patients (56%). Similar incident CRC case numbers and retrieval rates have been reported in molecular epidemiology studies embedded within other large cohorts. In two US studies on 901 and 1255 CRC cases, respectively, tissue specimens were retrieved from 58% and BRAF status available for 42% and 45% [30,31,38]. In a Swiss register on 1420 CRC patients, resection specimens were retrieved from 42% and BRAF analyses possible for 26% [28]. In these studies, no significant differences in characteristics were observed between patients whose tissue specimens could be retrieved or not, however only few prognostic factors were compared and a minority of the patients had stage IV disease. Among our mCRC patients, we found large differences as patients without available TMA had worse prognostic factors, received less treatment and had a shorter survival. Even for patients receiving chemotherapy, survival was shorter in patients without available TMA. In our study 91% of patients with a resection of the primary had available TMA compared to 46% of patients without a resection. In most instances, TMA cores can be sampled more easily utilizing surgical resection samples, whilst biopsy specimens, especially if small or necrotic, often do not yield enough tissue for processing. It is likely that surgery is less often performed in patients with higher tumor burden where PS is often affected. Our results question the generalizability of present TMA studies, and imply that we lack information on the biologically worst mCRC cases. In a recent study on 6155 randomized mCRC trial patients, tumor samples and BRAF analyses were available from 3063 patients (50%) [9]. The authors state that lack of available tissue is an important underexposed issue which may introduce sample bias in translational studies in mCRC. Our study clearly underlines this. To ensure the representativity of molecular studies more information is needed on patients without available TMA. Liquid biopsies with cell-free DNA analysis may in the future be a way to obtain molecular data from this large undescribed subgroup of mCRC patients.

In conclusion, BRAF mutations are more frequent in the general mCRC population than prior anticipated. Screening for BRAF mutations before selection of treatment strategy is therefore relevant for many patients, especially outside clinical trials. Our study shows a heterogenic median survival in the mCRC population and subgroups have an extremely short survival compared to trial patients. MutBRAF only partly explains the very poor prognosis of some mCRC patients and additional underlying molecular mechanisms may be involved. Patients without tumor tissue available for TMA and molecular analyses have more frequently poor prognostic characteristics, respond less to treatment and have a worse survival. Lack of available tissue is an important underexposed issue which introduces sample bias, and this must be recognized more clearly when conclusions are made from translational studies of mCRC trial patients.

## Supporting Information

S1 Table Frequency of BRAF mutations from metastatic colorectal cancer studies with > 200 patients. (DOCX)

S1 Appendix Data deposition. (XLS)

S2 Appendix Variables for data deposition. (PDF)
