# Supplementary material for: High BRAF Mutation Frequency and Marked Survival Differences in Subgroups According to KRAS/BRAF Mutation Status and Tumor Tissue Availability in a Prospective Population-Based Metastatic Colorectal Cancer Cohort
Source: PLoS One. 2015 Jun 29;10(6):e0131046. doi: 10.1371/journal.pone.0131046 (PMC4484806; doi:10.1371/journal.pone.0131046)
Supplement: S1 Table — (DOCX) [file pone.0131046.s001.docx]

| **S 1 Table. Frequency of BRAF mutations from metastatic colorectal cancer studies with > 200 patients.** | | | | | |
| --- | --- | --- | --- | --- | --- |
| Study | Authors | No patients | mutBRAF frequency | Colonic primary | mutBRAF in colon primary |
| CAIRO/CAIRO2/ COIN / FOCUS | Venderbosch S et al [9] | 3063 | 8.2 % | 70 % | 10.5 % |
|  | Yaeger R et al [33] | 1941 | 5 % |  |  |
| COIN | Maughan TS et al [5] | 1316 | 8 % | 70 % |  |
| CRYSTAL | van Cutsem E et al [1] | 999 | 6 % |  |  |
|  | Schirripa M et al [36] | 786 | 9 % | 70 % | 9.6% |
| FOCUS | Richman SD et al [21] | 711 | 7.9 % | 66 % |  |
| PRIME | Douillard JY et al [3] | 639 | 8 %* | 65 % |  |
| NORDIC 7 | Tveit KM et al [2] | 571 | 12 % | 59 % | 17.8 % |
|  | Tol J et al [42] | 559 | 8.7 % |  |  |
|  | Tran B et al [35] | 524 | 11 % |  |  |
|  | Saridaki Z et al [10] | 504 | 8.1 % | 72 % | 10 % |
|  | Morris VK et al [8] | 484 | 7.4 % | 69 % | 7.7 % |
| AGITG MAX | Price TJ et al [44] | 315 | 10.6 % | 77 % |  |
|  | Yokota T et al [6] | 229 | 6.5 % | 55 % | 17 % |
|  | Russo AL et al [7]. | 222 | 10 % | 71 % | 13.3 % |
|  | Loupakis F et al [24] | 214 | 7 % |  |  |
| PEAK | Schwartzberg LS et al [4] | 278 | 6 %* ^§^ | 68 % |  |
| FIRE-3 | Heinemann V et al [45] | 594 | 10 %*^§§^ | 61 % |  |
| Present study | Sorbye H et al | 446 | 20.6 % | 74 % | 27 % |
| *Among wildtype KRAS patients. ^§^Personal communication. ^§§^ In prior presentations: 48/ 488 patients. | | | | | |
