# Supplementary material for: High BRAF Mutation Frequency and Marked Survival Differences in Subgroups According to KRAS/BRAF Mutation Status and Tumor Tissue Availability in a Prospective Population-Based Metastatic Colorectal Cancer Cohort
Source: PLoS One. 2015 Jun 29;10(6):e0131046. doi: 10.1371/journal.pone.0131046 (PMC4484806; doi:10.1371/journal.pone.0131046)
Supplement: S2 Appendix — (PDF) [file pone.0131046.s003.pdf]

PatnoNew	None	Pat no + fixed number
Gender	{1, Male}...	
PrimTm	{1, Right colon}...	Location of primary
PrimTmRs	{1, Yes}...	Primary tumor resected
Liver	{0, no}...	Liver site of metastases
LymphNde	{0, no}...	Lymph node site of metastases
Lung	{0, no}...	Lung site of metastases
Peritnum	{0, no}...	Peritoneal site of metastases
NoOrgans	{1, 1}...	Number of organs involved
WHO	{0, 0}...	WHO performance status
WBC	None	White blood count at baseline
Platelet	None	Platelet count
AlkPhos	None	Alkaline phosphatase value
CEA	None	
CaPain	{0, 0}...	Cancer pain
WeigLoss	{0, < 5 %}...	Weigth loss last 3 months
Anorexi	{0, 0}...	Anorexi
Cmrbity1	{1, Yes}...	Comorbidity
StudTrtm	{1, Yes}...	Study treatment
BSC	{1, Yes}...	Best Supportive Care only
ChmF4	{1, Yes}...	Chemotherapy treated
Line1	{1, "yes"}...	1-line chemotherapy
FU1	{0, no}...	Chemotherapy given as 1-line
Oxalplt1	{0, no}...	
Irintec1	{0, no}...	
Bevaciz1	{0, no}...	
Cetuxim1	{0, no}...	
Line2	{1, "yes"}...	2-line chemotherapy
Line3	{1, "yes"}...	3.-line chemotherapy
CrtivSrg	{1, yes}...	Secondary surgery
PrimTmTodelt	{0, not colon}...	Pirmary tumor colon vs non-colon
minst1.ir.ox	{0, No}...	Combination chemotherapy given in 1-line
Linecount	None	Lines of chemotherapy given
Age	None	
AlkPhosNormal3	{0, > 3UNL}...	Alkaline phosphatase
Resp1F7	{1, CR}...	Response to 1-line chemotherapy
Resp2F7	{1, CR}...	Response to 2-line chemotherapy
Resp3F7	{1, CR}...	Response to 3.-line chemotherapy
BRAF	None	BRAF mutation status
MSI	None	MSI status
KRAS	None	KRAS mutation status
Smoker	{1, "Yes"}...	
Agedich	{0, <75}...	Age <75 vs >75
TMA	{0, "No TMA"}...	
BRAFnum	{0, wild}...	BRAF mutation status
KRASnum	{0, wild}...	
KRASdoublew	{0, doublewild}...	
NoOrgans2delt	{0, 1 organ}...	1 vs 2+ metastatic organs
synchronmet	{0, no}...	Synchronous metastatic disease
timetodeath	None	Overall survival in months

Prdemonths	None	PFS 1-line months
Prdemonths2	None	PFS 2-line
Prdemonths3	None	PFS 3-line
RASRAFtredelt	{1, mutBRAF}...	BRAF vs KRAS vs doblewild-type
WHO2delt	{0, 0}...	WHO 0 vs > 0
